# Are Sustainable and Healthy Eating Behaviors and Levels of Nutritional Knowledge Associated with Obesity and Anthropometric Indicators of Chronic Disease Risk Among Healthcare Workers?

**DOI:** 10.3390/healthcare14142142

**Published:** 2026-07-16

**Authors:** Hatice Baygut, Biriz Çakır

**Affiliations:** 1 Department of Nutrition and Dietetics, Faculty of Health Sciences, Suleyman Demirel University, 32260 Merkez, Isparta, Türkiye; 2 Department of Nutrition and Dietetics, Faculty of Health Sciences, Kırıkkale University, 71450 Yahşihan, Kırıkkale, Türkiye; birizcakir@kku.edu.tr

**Keywords:** anthropometric risk indicators, healthy eating behavior, healthcare workers, nutritional knowledge, obesity, sustainable eating

## Abstract

**Highlights:**

**What are the main findings?**

- Overweight, obesity, and anthropometric indicators associated with chronic disease risk were common among healthcare workers.
- Nutrition knowledge and sustainable healthy eating behaviors were strongly related; interpretation of their joint effects was restricted by high construct overlap. High nutrition knowledge also coexisted with a substantial prevalence of overweight and obesity.

**What are the implications of the main findings?**

- Workplace nutrition programs may address both nutrition knowledge and sustainable healthy eating behaviors among healthcare workers.
- Anthropometric risk screening can support targeted nutrition counseling in occupational health settings.

**Abstract:**

**Objectives:** This study examined sustainable and healthy eating behaviors, nutrition knowledge levels, obesity status, and anthropometric indicators associated with chronic disease risk among healthcare workers. **Methods:** A cross-sectional study was conducted with 1070 healthcare workers aged 22–59 years in Isparta, Türkiye. Data were collected using a face-to-face questionnaire, the Sustainable and Healthy Eating Behaviors Scale (SHE), the Nutrition Knowledge Level Scale for Adults (NKLSA), and anthropometric measurements. **Results:** Overall, 27.9% of participants were overweight and 27.1% had obesity. Anthropometric risk status was identified in 55.8% according to waist circumference, 54.8% according to neck circumference, 55.0% according to waist-to-hip ratio, and 70.2% according to waist-to-height ratio. The mean SHE score was 126.53 ± 61.65. SHE scores decreased across BMI groups from underweight to obesity (189.37 ± 20.07 to 105.70 ± 56.81; *p* < 0.001). SHE was strongly correlated with NKLSA (r = 0.955; 95% CI: 0.950–0.960) and negatively correlated with BMI (r = −0.333), waist circumference (r = −0.211), waist-to-hip ratio (r = −0.230), and waist-to-height ratio (r = −0.196). **Conclusions:** Nutrition knowledge, sustainable healthy eating behavior, and anthropometric risk indicators were closely interrelated among healthcare workers. High nutrition knowledge scores coexisted with a high burden of overweight, obesity, and anthropometric risk, suggesting a gap between knowledge and body-weight status that may be shaped by occupational and lifestyle-related factors.

## 1. Introduction

Obesity, undernutrition, and climate change represent a “*global syndemic*” affecting societies worldwide because these conditions co-occur across time and settings, interact to produce complex outcomes, and share common underlying societal drivers [1]. In 2022, 43% of adults aged 18 years and older worldwide were overweight, and 16% had obesity. In addition, more than 390 million children and adolescents aged 5–19 years were overweight, among whom 160 million were living with obesity [2]. In addition to obesity, noncommunicable diseases (NCDs), also referred to as chronic diseases, including cardiovascular diseases, cancer, diabetes, and chronic respiratory diseases, are highly prevalent and account for 80% of premature deaths related to chronic diseases worldwide. Unhealthy diet, physical inactivity, tobacco use, harmful alcohol consumption, and air pollution are recognized as major risk factors for these diseases [3]. Climate change, through extensive effects on human and planetary health, further intensifies these global health challenges [1]. In this context, sustainable nutrition has gained increasing importance in achieving several United Nations Sustainable Development Goals aimed at protecting planetary health, eliminating poverty, and ensuring health and well-being for all [4].

The Food and Agriculture Organization of the United Nations (FAO) defines sustainable nutrition as a dietary approach with low environmental impact that contributes to food and nutrition security and healthy living for present and future generations, protects biodiversity and ecosystems, is culturally acceptable, accessible, economically fair and affordable, nutritionally adequate, safe, and healthy, and optimizes natural and human resources [5]. In line with this approach, the EAT-Lancet Commission recommends reducing consumption of foods associated with high environmental costs, particularly red meat and sugar, by at least 50%, while increasing consumption of plant-based foods such as fruits, vegetables, and legumes by more than 100% [6]. Healthy eating patterns are increasingly evaluated together with planetary health, reflecting the close relationship between dietary patterns and food systems [7]. Accordingly, sustainable healthy diets are defined as dietary patterns that promote all dimensions of health and well-being, exert low environmental pressure and impact, and are accessible, affordable, safe, equitable, and culturally acceptable [6].

In addressing the global syndemic, healthcare workers are expected not only to promote healthy eating but also to advocate sustainable and healthy dietary patterns by considering planetary health. Because of high credibility and close contact with the general population, healthcare workers may serve as agents of change in promoting sustainable dietary practices, particularly given that current dietary habits are not sustainable in many countries [8]. However, studies have reported insufficient knowledge regarding healthy and sustainable nutrition among healthcare personnel [8,9], and interventions targeting knowledge, attitudes, and behaviors may improve alignment with planetary health goals [10].

Obesity and chronic diseases also affect healthcare personnel. Previous studies have reported high rates of overweight and obesity among healthcare workers in the United States and obesity among nurses in the United Kingdom [11,12]. Chronic diseases are also common among healthcare workers. Studies have reported that approximately one-third of healthcare workers in Qatar had at least one chronic disease [13], whereas varying rates of chronic disease and hypertension have been identified among healthcare workers in sub-Saharan countries [14]. In Türkiye, 36.1% of healthcare workers were reported to have at least one diagnosed disease, including cardiovascular diseases, diabetes, hypertension, and cancer [15]. The prevalence of obesity and chronic diseases among healthcare workers varies according to factors such as dietary habits, alcohol consumption, insufficient physical activity, sleep patterns, occupation, working environment, and job-related stress [16,17,18]. Dietary models aligned with planetary health goals among healthcare workers are relevant for public health and for the health profile of healthcare workers themselves, who serve as role models for society.

This study was conducted to evaluate sustainable and healthy eating behaviors and levels of nutritional knowledge in relation to obesity and anthropometric indicators of chronic disease risk among healthcare workers.

## 2. Materials and Methods

### 2.1. Subjects

This cross-sectional study was conducted in Isparta, Türkiye, between June and October 2025. Healthcare workers employed in healthcare centers located in the central districts of Isparta province were selected using a random sampling method. The sampling frame comprised eligible staff lists available at participating institutions. Clear information regarding the purpose of the study was provided to healthcare workers, and individuals who voluntarily agreed to participate were included. Individuals with psychological disorders, those using psychotherapeutic medications, pregnant individuals, those following a weight loss program, and individuals who did not volunteer to participate were excluded from the study. Data were collected using a face-to-face questionnaire. The questionnaire included items related to demographic characteristics of healthcare workers, including age, marital status, and occupation. Occupational groups were classified as physicians/specialist physicians, nurses, health professionals with a four-year undergraduate degree, and health technicians with a two-year associate degree. Field records contained completed questionnaires only; a formal response-rate denominator was unavailable. Based on the a priori power analysis conducted using G*Power software, version 3.1.9.7 (Heinrich-Heine-Universität Düsseldorf, Düsseldorf, Germany), the minimum required sample size was estimated to be 424 participants, assuming a statistical power of 95%. A total of 1070 healthcare workers aged 22–59 years voluntarily participated in the study.

### 2.2. Measures

#### 2.2.1. Anthropometric Measurements

Anthropometric measurements were obtained from participants through face-to-face assessments. Body weight was measured in kilograms using an electronic scale, and height was measured using a stadiometer. Body weight was obtained using BC-545N InnerScan Segmental Body Composition Monitor (Tanita Corporation, Tokyo, Japan); TANITA-derived body-composition outputs were outside the prespecified analytic endpoint set. Body mass index (BMI) was calculated using the formula body weight/height squared (kg/m^2^) and classified into four categories according to World Health Organization criteria: underweight (<18.5 kg/m^2^), normal weight (18.5–24.9 kg/m^2^), overweight (25.0–29.9 kg/m^2^), and obesity (≥30.0 kg/m^2^) [19]. Waist circumference (WC) and hip circumference (HC) were measured in accordance with the measurement procedures described in the WHO report. Increased waist circumference and waist-to-hip ratio (WHR) were treated as anthropometric indicators associated with chronic disease risk. The cut-off values were defined as ≥80 cm for women and ≥94 cm for men for waist circumference, and ≥0.85 for women and ≥0.90 for men for WHR [20]. Waist-to-height ratio (WHtR) was classified as normal/no risk at 0.4–<0.5 and as risk present at <0.4 or ≥0.5. Neck circumference (NC), which is associated with cardiometabolic risk factors, was also assessed. Neck circumference cut-off values of ≥37 cm for men and ≥33 cm for women were used to evaluate this risk factor [21].

#### 2.2.2. The Sustainable and Healthy Eating Behaviors Scale (SHE)

The Sustainable and Healthy Eating Behaviors Scale (SHE) was developed on the basis of the Food and Agriculture Organization’s definition of sustainable diets, the LiveWell approach, and principles of sustainable and healthy eating habits. The scale was first developed by Żakowska-Biemans et al. in 2019 and originally consisted of 8 factors and 34 items [22]. The Turkish adaptation was conducted by Köksal et al. in 2023 [23], resulting in the Sustainable and Healthy Eating Behaviors Scale. The Turkish version consists of 7 factors and 32 items. These seven factors are quality labels (local and organic), seasonal foods and avoidance of food waste, animal welfare, reduced meat consumption, healthy and balanced eating, local food, and low-fat consumption. The scale is a 7-point Likert-type instrument with responses scored as follows: 1 = never, 2 = very rarely, 3 = rarely, 4 = sometimes, 5 = often, 6 = very often, and 7 = always. Both subscale scores and a total score are calculated. The theoretical total score range is 32–224. Higher total scores indicate higher levels of sustainable and healthy eating behaviors [23].

#### 2.2.3. Nutrition Knowledge Level Scale (NKLSA)

The NKLSA was used to determine participants’ nutrition knowledge levels. The scale was developed by Batmaz in 2018, and validity and reliability were established. The scale consists of two sections. The first section includes 32 items, comprising 20 items related to basic nutrition and 12 items related to food preferences. All items in these two sections are evaluated using a 5-point Likert scale with the following response options: strongly agree, agree, neither agree nor disagree, disagree, and strongly disagree. For correct statements, responses are scored as follows: strongly agree = 4 points, agree = 3 points, neither agree nor disagree = 2 points, disagree = 1 point, and strongly disagree = 0 points. Reverse scoring is applied for incorrect statements: strongly agree = 0 points, agree = 1 point, neither agree nor disagree = 2 points, disagree = 3 points, and strongly disagree = 4 points. The maximum possible score is 80 points for the basic nutrition section and 48 points for the food preferences section. Based on scale scores, participants’ nutrition knowledge is classified as poor, moderate, good, or very good. In the basic nutrition section, scores of <45 are classified as poor, 45–55 as moderate, 56–65 as good, and >65 as very good. In the food preferences section, scores of <30 are classified as poor, 30–36 as moderate, 37–42 as good, and >42 as very good [24].

### 2.3. Data Audit and Statistical Analysis

Statistical analyses were performed using R (version 4.4.2) and Python (version 3.12). Descriptive statistics were reported as n (%), mean ± standard deviation, and 95% confidence intervals where appropriate. Group comparisons used chi-square tests, Student’s *t*-test, and one-way ANOVA with Tukey HSD post hoc procedures. ANOVA assumptions were examined using residual diagnostics and Levene tests. Effect-size estimates were reported using eta-squared and omega-squared for ANOVA models and Cohen’s d for two-group comparisons (Supplementary Tables S8 and S9). Analyses used available records for each variable set, without imputation. SHE scores were checked against theoretical score ranges before interpretation. The original dataset included 88 observations below the theoretical SHE minimum and 2 observations with a total SHE score of zero. Sensitivity analyses were conducted after excluding observations outside the theoretical SHE range. Correlation analyses included Pearson coefficients with 95% confidence intervals. Collinearity diagnostics were examined using variance inflation factors (VIF). Regression models used HC3 robust standard errors. The path model was treated as a cross-sectional indirect association model using the lavaan package in R; saturated-model fit indices were reported as descriptive model information. Direct and indirect paths with opposite signs were classified as an inconsistent mediation/suppression pattern. Internal consistency was examined at the subscale/section-score level because item-level responses were not available in the analysis file; SHE estimates were based on the seven subscale scores, and NKLSA estimates were based on the two section scores. These coefficients were therefore interpreted as score-level consistency indices rather than item-level reliability coefficients.

## 3. Results

**Figure 1:**
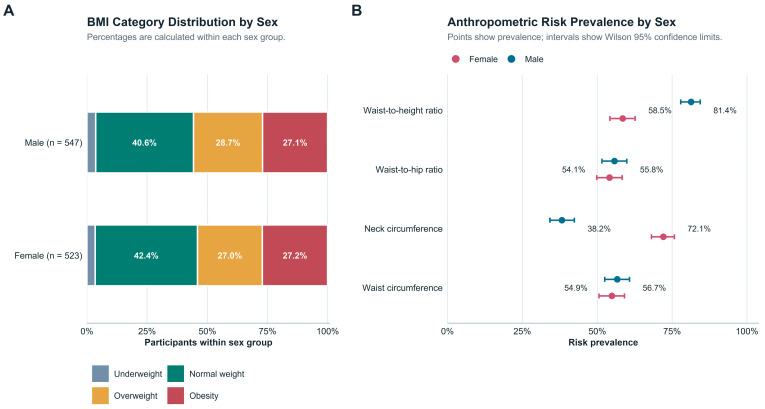
Anthropometric risk profile of healthcare workers. (**A**) BMI category distribution by sex. (**B**) Sex-specific prevalence of anthropometric indicators associated with chronic disease risk; points show percentages and intervals show Wilson 95% confidence limits.

In this study, 48.87% of healthcare workers were women and 51.12% were men; 52.20% were aged 30–49 years, and 62.1% were married. The highest participation rate was observed among health professionals with a four-year undergraduate degree (30.70%), whereas physicians had the lowest participation rate (21.90%). Overweight was identified in 27.90% of healthcare workers and obesity in 27.10%, with no statistically significant difference between sexes (*p* = 0.908). Anthropometric risk status was identified in 55.80% of healthcare workers according to waist circumference (women: 54.9%; men: 56.70%), 54.80% according to neck circumference (women: 72.10%; men: 38.20%), 55.0% according to waist-to-hip ratio (women: 54.10%; men: 55.80%), and 70.2% according to waist-to-height ratio (women: 58.5%; men: 81.4%). Evaluation of anthropometric risk indicators demonstrated no significant sex-based difference according to waist circumference and waist-to-hip ratio (*p* > 0.05). Statistically significant differences between sexes were observed according to neck circumference and waist-to-height ratio (*p* < 0.001) (Table 1; Figure 1A,B).

Before interpreting SHE and NKLSA score distributions, internal consistency was examined at the subscale/section-score level. Internal consistency was high for SHE in both the original dataset and the theoretical-range-screened dataset (Cronbach’s α = 0.970; McDonald’s ω = 0.999 for both). NKLSA also showed high section-score-level consistency in the original dataset (α = 0.939; ω = 0.999) and within the SHE theoretical-range-screened dataset (α = 0.931; ω = 0.998). These estimates reflect consistency across subscale or section scores.

Evaluation of anthropometric measurements among healthcare workers demonstrated higher waist circumference and hip circumference values in men than in women (*p* < 0.001) (Table 2).

The total SHE score of healthcare workers was calculated as 126.53 ± 61.65 (men: 129.58 ± 61.03; women: 123.33 ± 62.19), and no statistically significant sex-based difference was found in either the total score or subdimension scores (*p* > 0.05). The theoretical-range audit identified 982 observations within the valid SHE total range; the sensitivity dataset had a mean SHE total score of 136.38 ± 54.35. Similarly, no significant difference was observed between women and men in the mean NKLSA scores of healthcare workers (*p* > 0.05) (Table 3; Supplementary Tables S1–S3).

The distribution of healthcare workers’ scores for the NKLSA subdimensions showed that most participants had “very good” knowledge levels in both basic nutrition knowledge and food preference knowledge. Among individuals with “very good” basic nutrition knowledge and food preference knowledge, the number of men was higher than the number of women in both subdimensions (M: 315, F: 273; M: 315, F: 273, respectively). Individuals with “poor” knowledge levels ranked second in both subdimensions, with similar numbers of women and men (M: 145, F: 151; M: 153, F: 156, respectively).

In other words, 55% of healthcare workers scored at the “very good” level in the basic nutrition knowledge subdimension, whereas 27.7% scored at the “poor” level. This distribution did not differ significantly between women and men (*p* = 0.102). Similarly, in the food preference subdimension, 55% of healthcare workers demonstrated “very good” food preference knowledge, whereas 28.9% were classified in the “poor” category. No statistically significant sex-based difference was found in food preference knowledge (*p* = 0.070) (Table 3).

**Figure 2:**
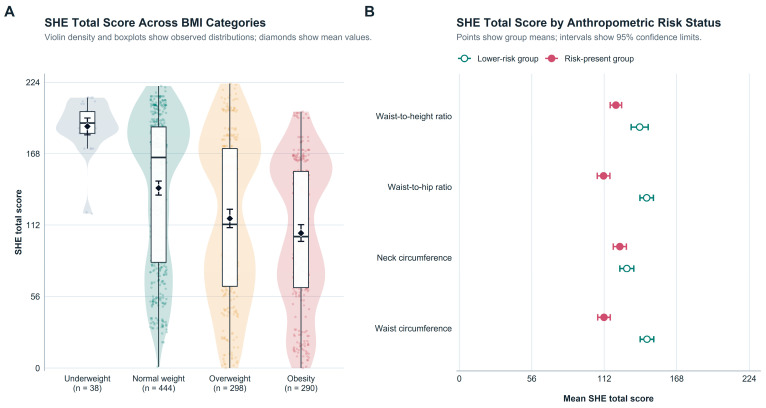
Sustainable and Healthy Eating Behaviors Scale (SHE) total score by obesity and anthropometric risk groups. (**A**) SHE total score across BMI categories; violin densities and boxplots show observed distributions, and diamonds show means. (**B**) Mean SHE total score by anthropometric risk status; points show means and intervals show 95% confidence limits.

Evaluation of the total score and all subdimension scores obtained from the SHE according to BMI groups among healthcare workers demonstrated statistically significant differences between groups (*p* < 0.001 for all subdimensions). As BMI increased, the total SHE score decreased. The highest total SHE score was observed among underweight healthcare workers (189.37 ± 20.07), followed by individuals with normal weight (141.02 ± 58.87), overweight (117.18 ± 63.17), and obesity (105.70 ± 56.81). Distributional differences across BMI categories are shown in Figure 2A. In the subdimensions of “quality labels (local and organic),” “seasonal foods and avoidance of food waste,” and “healthy and balanced eating,” underweight healthcare workers had higher scores than individuals in the other BMI groups, and these differences were statistically significant (*p* < 0.001). According to post hoc analysis results, all BMI groups differed significantly from one another in some subdimensions, whereas similar scores were observed particularly between the overweight and obesity groups in several subdimensions. Differences between nutrition knowledge subdimension scores and BMI groups were also statistically significant (*p* < 0.05) (Table 4).

In comparisons based on anthropometric indicators among healthcare workers, lower total SHE scores were observed in risk-present groups according to waist circumference (111.84 ± 60.38 vs. 145.06 ± 58.21), waist-to-hip ratio (111.52 ± 60.34 vs. 144.83 ± 58.25), and waist-to-height ratio (121.05 ± 61.65 vs. 139.42 ± 59.82) (*p* < 0.001). The neck circumference comparison was smaller and not statistically significant. Mean SHE differences by anthropometric risk status are summarized in Figure 2B. Similarly, in the NKLSA, the risk groups had lower scores in both the basic nutrition and food preference subdimensions. A poor level of basic nutrition score was significantly more common in the risk group (*p* = 0.031) (Table 5).

When healthcare workers were examined according to BMI groups, poor nutrition knowledge in both subdimensions was more common among overweight individuals, whereas moderate knowledge levels were higher among individuals with obesity (*p* < 0.001). Table 6 includes all BMI categories, including underweight and normal-weight groups. Healthcare workers without risk status according to waist circumference and waist-to-hip ratio had a statistically significantly higher rate of “very good” basic nutrition and food preference knowledge compared with those at risk (*p* < 0.001). Individuals with normal waist-to-height ratio and those in the <0.4 risk group showed higher “very good” knowledge proportions than those in the ≥0.5 risk group (*p* < 0.001). Neck circumference risk groups showed comparable basic nutrition and food preference knowledge distributions (*p* = 0.503) (Table 6; Supplementary Table S10).

**Figure 3:**
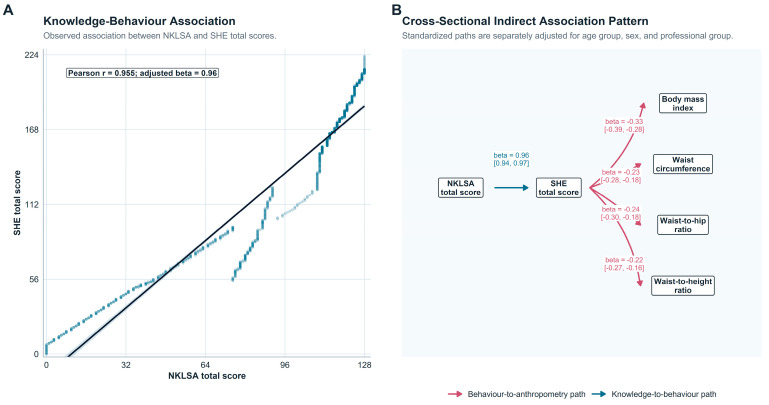
NKLSA–SHE overlap and cross-sectional indirect association pattern. (**A**) Relationship between NKLSA total and SHE total scores; density-based scatter display summarizes overlapping observations. (**B**) Standardized adjusted behavior-to-anthropometry paths; Table 7 presents unstandardized path coefficients for the same cross-sectional indirect association structure.

Correlation analysis showed a very strong positive association between SHE and NKLSA scores (r = 0.955; *p* < 0.001; 95% CI: 0.950 to 0.960). The theoretically consistent SHE score set yielded a comparable coefficient (r = 0.948), reflecting close overlap between nutrition knowledge and self-reported sustainable healthy eating behavior. SHE scores were negatively correlated with BMI (r = −0.333; *p* < 0.001), waist circumference (r = −0.211; *p* < 0.001), waist-to-hip ratio (r = −0.230; *p* < 0.001), and waist-to-height ratio (r = −0.196; *p* < 0.001). Models including NKLSA and SHE together showed elevated VIF values; coefficients from these joint models represent conditional estimates within the same model structure (Figure 3A; Supplementary Tables S4 and S5). Together with the score-level consistency estimates, this correlation suggests that NKLSA and SHE may not have separated clearly as independent constructs in this sample.

Cross-sectional path analysis summarized direct and indirect association patterns between NKLSA, SHE, and anthropometric indicators. The saturated path model yielded CFI = 0.998, TLI = 0.987, RMSEA = 0.001, and SRMR < 0.001; these indices are presented as descriptive model information. NKLSA was strongly associated with SHE (β = 0.955), explaining 91.3% of SHE variance. Direct and indirect paths showed opposite directions for BMI, waist circumference, waist-to-hip ratio, and waist-to-height ratio, forming an inconsistent indirect association/suppression pattern. In the BMI model, the NKLSA direct path was positive (c′ = 1.025), whereas the SHE path was negative (b = −1.312), yielding a negative indirect path (a × b = −1.254). The neck circumference model yielded a non-significant indirect path (Table 7; Figure 3B; Supplementary Table S6).

## 4. Discussion

This study examined sustainable and healthy eating behaviors, nutrition knowledge levels, obesity status, and anthropometric indicators associated with chronic disease risk among healthcare workers.

The literature indicates that overweight and obesity are highly prevalent among healthcare workers. Although these conditions are reported more frequently in Western countries such as the United States and the United Kingdom [11,12], high prevalence rates have also been identified in China [17], Saudi Arabia [25], Nigeria [26], and Türkiye [15,27]. Consistent with these findings, overweight and obesity were identified in 55.0% of healthcare workers. This high prevalence may be associated with mental stress, sociodemographic and occupational characteristics, health-related factors, and working conditions [17,28]. In addition, metabolic syndrome, obesity, and hypertension, which are major risk factors for type 2 diabetes, cardiovascular diseases, stroke, and cancer, are common among healthcare workers worldwide [29].

Anthropometric indicators are practical tools for estimating cardiometabolic risk and predicting metabolic disorders [30,31]. Waist circumference, neck circumference, waist-to-hip ratio, and waist-to-height ratio were evaluated, and approximately half of healthcare workers had risk status according to several anthropometric indicators. Waist-to-height ratio classified 70.2% of participants as having risk present when the <0.4 or ≥0.5 criterion was applied. Waist circumference and waist-to-hip ratio showed comparable risk status by sex, whereas neck circumference risk status was higher among women and waist-to-height ratio risk status was higher among men (Table 1; Figure 1B). Neck circumference reflects upper-body subcutaneous adiposity, whereas waist circumference is an indicator of visceral adiposity [31,32]. The high neck-circumference risk rate among women is consistent with possible sex- and population-related variation in NC cut-off performance. Sex-based differences in fat distribution may also contribute to these findings, with central and visceral fat accumulation more common in men and total, lower-body, and subcutaneous fat accumulation generally higher in women [31,33].

Waist circumference is widely used in clinical practice, and elevated waist circumference has been associated with increased risk of hypertension, type 2 diabetes, hypercholesterolemia, joint pain, low back pain, and hyperuricemia [34]. Although hip circumference is often overlooked because it is primarily considered a component of waist-to-hip ratio, it has also been associated with adverse health outcomes [35]. In this study, waist and hip circumference values were higher in men than in women (Table 2). Although higher waist circumference in men is consistent with android-type obesity and visceral fat accumulation, the higher hip circumference observed in men may be associated with the higher prevalence of overweight and obesity, larger skeletal structure, and greater muscle mass among male participants [36].

Lifestyle factors, particularly nutrition, physical activity, smoking, and alcohol consumption, are known to play important roles in reducing obesity and anthropometric risk indicators [3]. From the perspective of planetary health, sustainable dietary patterns represent a relevant component of health promotion [37]. Healthcare workers have a key role in promoting sustainable food systems and improving both health outcomes and environmental sustainability [38]. Workplace interventions can combine nutrition education, sustainable menu planning, and environmental arrangements that make healthier choices easier during work routines [39].

Determining knowledge levels related to sustainable nutrition is essential for promoting sustainable dietary behaviors [40]. Previous studies have reported that health sciences students and healthcare workers may have higher knowledge levels than the general population, whereas insufficient knowledge and negative attitudes are associated with higher consumption of red and processed meat [39]. Other studies have shown that some healthcare workers have never heard the term “sustainable diet” and that most possess limited knowledge, although this topic is considered important and willingness to receive training is high [8]. In addition, dietary quality and sustainability may decline during night shifts among healthcare workers engaged in shift work [40]. In the present study, the mean total score for sustainable and healthy eating behaviors was 126.53 ± 61.65, indicating a moderate level, and these behaviors did not differ significantly by sex (Table 3). After theoretical-range screening, the mean SHE total score was 136.38 ± 54.35, supporting the same moderate-score interpretation with a narrower valid-score distribution.

Several studies have examined nutrition knowledge among healthcare workers [41,42,43]. Although healthcare workers may possess better nutrition knowledge than individuals outside the healthcare sector [44], knowledge gaps persist among medical students, physicians, general practitioners, nurses, and other healthcare professionals [41,42,43,45]. In this study, 55% of healthcare workers demonstrated “very good” levels of both basic nutrition knowledge and food preference knowledge, whereas 27.7% had “poor” basic nutrition knowledge and 28.9% had “poor” food preference knowledge. Nutrition knowledge did not differ significantly by sex (Table 3). The coexistence of very good and poor knowledge categories indicates heterogeneity in nutrition-related knowledge within the healthcare workforce.

Sustainable and healthy eating behavior was highest among underweight participants and lowest among individuals with obesity. Underweight healthcare workers demonstrated higher scores in the subdimensions of “quality labels,” “seasonal foods and avoidance of food waste,” and “healthy and balanced eating” compared with the other BMI groups (Table 4; Figure 2A). This finding may be associated with greater attention to body weight control and healthier food choices. Sustainable and healthy dietary patterns characterized by higher consumption of vegetables, fruits, whole grains, and fiber and lower energy density are effective in preventing malnutrition and reducing the risk of obesity and chronic diseases [46]. Post hoc analyses demonstrated that some BMI groups differed significantly across subdimensions, whereas the overweight and obesity groups showed similar scores in certain areas, possibly because of similar dietary and physical activity habits.

Sustainable dietary patterns are important for obesity prevention and for lower levels of anthropometric indicators linked to chronic disease risk [46]. In this study, healthcare workers with risk present according to waist circumference, waist-to-hip ratio, and waist-to-height ratio had lower sustainable and healthy eating behavior scores (Table 5; Figure 2B). Similarly, risk groups demonstrated lower scores in both the basic nutrition knowledge and food preference knowledge subdimensions of the Nutrition Knowledge Level Scale. These findings indicate that sustainable and healthy dietary behaviors are closely related to anthropometric risk profiles among healthcare workers.

Although nutrition knowledge is important, the translation of knowledge into attitudes and behaviors is central in knowledge, attitude, and practice studies [44,47]. In this study, nearly half of healthcare workers with overweight and obesity demonstrated “very good” levels of basic nutrition knowledge and food preference knowledge; knowledge level alone did not align consistently with lower body weight status (Table 6). Workload, shift work, stress, sleep quality, physical activity, and food availability during work hours may influence the gap between knowledge and behavior. Nutrition and health knowledge are associated with dietary behaviors and health outcomes, yet organizational conditions may shape the degree to which knowledge is reflected in daily choices [48]. Thus, nutrition knowledge was present in many participants, yet overweight and obesity remained common, indicating that knowledge alone did not correspond to maintenance of an appropriate body-mass profile in this occupational group.

In the present study, except for neck circumference, the proportion of healthcare workers with “very good” nutrition knowledge was higher among individuals without risk according to waist circumference and waist-to-hip ratio and among individuals with normal waist-to-height ratio or waist-to-height ratio <0.4 (Table 6). Nutrition knowledge and sustainable and healthy eating behavior were very strongly correlated (r = 0.955; *p* < 0.001). The magnitude of this association remained high after SHE theoretical-range screening and indicated possible construct overlap, common-method variance, and collinearity. Higher nutrition knowledge was associated with lower BMI, waist circumference, and waist-to-hip ratio in bivariate analyses (Figure 3A; Supplementary Tables S4 and S5). At the score level, the high α/ω estimates and the r = 0.955 association suggest a tightly coupled knowledge-behavior response pattern, with limited empirical separation between NKLSA and SHE in this sample.

A cross-sectional path model was used to examine direct and indirect association patterns. The model demonstrated a very strong positive relationship between nutrition knowledge and sustainable and healthy eating behaviors (β = 0.955), explaining 91.3% of the variance in SHE. Saturated-model fit indices were treated descriptively and were not used as evidence of superior model validity. For BMI, waist circumference, waist-to-hip ratio, and waist-to-height ratio, direct and indirect paths had opposite signs. This pattern was classified as inconsistent mediation/suppression. Neck circumference showed no statistically significant indirect association pattern (Table 7; Figure 3B). In line with the cross-sectional design and the strong NKLSA–SHE overlap, the model describes conditional associations among NKLSA, SHE, and anthropometric indicators.

## 5. Conclusions

In this study, overweight and obesity were common among healthcare workers, affecting 55.0% of participants regardless of sex. Anthropometric risk status varied according to the indicator used. Waist circumference and waist-to-hip ratio demonstrated no sex-based difference, whereas risk assessed by neck circumference and waist-to-height ratio differed by sex. Waist and hip circumference values were higher in men than in women. Although the study was conducted in southern Türkiye, where the Mediterranean diet is traditionally common, sustainable and healthy eating behaviors among healthcare workers remained at a moderate level. Nutrition knowledge levels were heterogeneous. Although the largest group demonstrated “very good” knowledge, individuals with “poor” knowledge constituted the second largest group. The presence of “very good” nutrition knowledge among nearly half of healthcare workers with overweight and obesity indicates that knowledge alone may not align with healthy body weight status. Nutrition knowledge, sustainable healthy eating behavior, and anthropometric adiposity indicators were closely interrelated among healthcare workers. The very high NKLSA–SHE correlation showed close overlap between knowledge and self-reported eating behavior scores. Workplace nutrition education, sustainable menu planning by dietitians, and work-environment arrangements that facilitate healthier choices may support the health of healthcare workers and public health.

## 6. Limitations

This study has several limitations. The cross-sectional design limits temporal and causal inference. Because the study was conducted among volunteer healthcare workers in a provincial center in Türkiye, generalizability to all healthcare workers is limited. Field logs contained completed questionnaires only; the absence of a formal refusal denominator limits response-rate estimation. The analytic endpoint set included anthropometric measurements and circumference-based ratios; TANITA-derived body-composition outputs were outside the endpoint set. The SHE audit identified observations outside the theoretical score range; sensitivity analyses were included to evaluate the influence of these observations. The very high correlation between NKLSA and SHE indicates possible construct overlap and common-method variance, and coefficients from models including both constructs are conditional estimates. Physical activity, shift work, sleep quality, smoking, alcohol use, and clinical/laboratory indicators were not available in the analytic dataset. One strength of this study is that the sample was not limited to a single occupational group but included healthcare workers from different professions. Anthropometric measurements were obtained using standardized methods by trained interviewers after appointments had been scheduled at times convenient for participants. Larger multicenter studies with physical activity, shift work, sleep quality, smoking, alcohol use, and clinical/laboratory indicators can provide more detailed evaluation of nutrition-related anthropometric risk profiles.

## Figures and Tables

**Table 1:** Distribution of demographic characteristics and obesity and anthropometric risk status determined according to anthropometric parameters by sex among healthcare workers.

	Female (n = 523)		Male (n = 547)		Total (n = 1070)		*p*
	n	%	n	%	n	%	
**Demographic Characteristics**							
**Age (year)**							
22–29	111	21.20	134	24.50	245	22.90	0.377
30–49	283	54.10	276	50.50	559	52.20	
50–59	129	24.70	137	25.00	266	24.90	
**Marital Status**							
Married	324	62.00	341	62.30	665	62.10	0.895
Single	199	38.00	206	37.70	405	37.90	
**Occupations**							
Physician/Specialist	114	21.80	120	21.90	234	21.90	0.475
Nurse	124	23.70	128	23.40	252	23.60	
Health Professionals with a Four-Year Undergraduate Degree	170	32.50	159	29.10	329	30.70	
Health Technicians with a Two-Year Associate Degree	115	22.00	140	25.60	255	23.80	
**BMI (kg/m^2^)**							
Underweight	18	3.40	20	3.70	38	3.60	0.908
Normal Weight	222	42.40	222	40.60	444	41.50	
Overweight	141	27.00	157	28.70	298	27.90	
Obesity	142	27.20	148	27.10	290	27.10	
**Anthropometric risk status**							
**Waist circumference (cm)**							
No risk	236	45.10	237	43.30	473	44.20	0.554
Risk present	287	54.90	310	56.70	597	55.80	
**Neck circumference (cm)**							
No risk	146	27.90	338	61.80	484	45.20	<0.001 *
Risk present	377	72.10	209	38.20	586	54.80	
**Waist-to-hip ratio**							
No risk	240	45.90	242	44.20	482	45.00	0.588
Risk present	283	54.10	305	55.80	588	55.00	
**Waist-to-height ratio**							
No risk (0.4–<0.5)	217	41.5	102	18.6	319	29.8	<0.001 *
Risk present (<0.4)	40	7.6	0	0	40	3.7	
Risk present (≥0.5)	266	50.9	445	81.4	711	66.4	

*: Significant at the 0.05 level according to chi-square analysis. BMI: underweight: <18.5 kg/m^2^, normal: 18.5–24.9 kg/m^2^, overweight: 25.0–29.9 kg/m^2^, obesity: ≥30.0 kg/m^2^ For waist circumference: No risk: women < 80 cm, men < 94 cm; Risk present: women ≥ 80 cm, men ≥ 94 cm For neck circumference: No risk: women < 33 cm, men < 37 cm; Risk present: women ≥ 33 cm, men ≥ 37 cm For waist-to-hip ratio: No risk: women < 0.85, men < 0.90; Risk present: women ≥ 0.85, men ≥ 0.90 For waist-to-height ratio: For women and men: Normal/No risk: 0.4–<0.5; Risk present: <0.4 or ≥0.5.

**Table 2:** Distribution of mean and standard deviation values of anthropometric measurements and BMI classification by sex among healthcare workers.

	Female (n = 523)	Male (n = 547)	Total (n = 1070)	*p*
	Mean ± SD	Mean ± SD	Mean ± SD	
**Anthropometric measurements**				
Body weight (kg)	71.13 ± 17.22	71.56 ± 16.47	71.34 ± 16.83	0.676
Height (cm)	165.14 ± 6.24	165.71 ± 5.96	165.43 ± 6.10	0.124
Waist circumference (cm)	83.65 ± 14	97.57 ± 14.82	90.76 ± 16	<0.001 *
Hip circumference (cm)	97.62 ± 11.27	106.58 ± 10.8	102.19 ± 11.90	<0.001 *
Neck circumference (cm)	35.99 ± 4.39	35.91 ± 4.18	35.95 ± 4.28	0.762
**BMI (kg/m^2^)**				
Underweight	17.95 ± 0.46	17.71 ± 0.70	17.82 ± 0.60	0.217
Normal weight	21.51 ± 1.26	21.49 ± 1.30	21.50 ± 1.28	0.862
Overweight	26.26 ± 1.15	26.31 ± 1.12	26.29 ± 1.14	0.688
Obesity	33.46 ± 2.59	33.28 ± 2.12	33.37 ± 2.36	0.520

*: Significant at the 0.05 level according to Student’s *t*-test. BMI: underweight: <18.5 kg/m^2^, normal: 18.5–24.9 kg/m^2^, overweight: 25.0–29.9 kg/m^2^, obesity: ≥30.0 kg/m^2^.

**Table 3:** Distribution of Sustainable and Healthy Eating Behaviors Scale (SHE) scores and Nutrition Knowledge Level Scale (NKLSA) scores by sex among healthcare workers.

	Female (n = 523)	Male (n = 547)	Total (n = 1070)	*p*
	Mean ± SD	Mean ± SD	Mean ± SD	
**SHE subdimensions**				
1. Quality labels (local and organic)	30.92 ± 15.60	32.42 ± 15.28	31.69 ± 15.45	0.112
2. Seasonal foods and avoidance of food waste	27.02 ± 13.64	28.38 ± 13.32	27.71 ± 13.49	0.100
3. Animal welfare	15.48 ± 7.78	16.27 ± 7.61	15.88 ± 7.7	0.094
4. Reduced meat consumption	11.56 ± 5.85	12.22 ± 5.76	11.9 ± 5.81	0.062
5. Healthy and balanced eating	15.42 ± 7.77	16.21 ± 7.68	15.82 ± 7.73	0.094
6. Local food	11.46 ± 5.84	12.07 ± 5.74	11.77 ± 5.8	0.082
7. Low-fat consumption	11.48 ± 5.84	12.01 ± 5.75	11.75 ± 5.8	0.129
Total score	123.33 ± 62.19	129.58 ± 61.03	126.53 ± 61.65	0.097
**NKLSA**				
**Basic Nutrition Knowledge**				
Poor	22.30 ± 14.42	22.70 ± 13.83	22.50 ± 14.11	0.808
Moderate	50.47 ± 2.73	50.64 ± 3.03	50.54 ± 2.86	0.705
Good	60.75 ± 3.49	61 ± 2.98	60.90 ± 3.10	0.861
Very good	73.24 ± 4.10	73.05 ± 4.37	73.14 ± 4.24	0.600
**Food Preference**				
Poor	15.20 ± 9.84	15.77 ± 9.58	15.48 ± 9.70	0.609
Moderate	33.63 ± 1.65	33.87 ± 1.67	33.74 ± 1.65	0.386
Good	39.75 ± 1.66	40 ± 1.51	39.90 ± 1.54	0.724
Very Good	45.47 ± 1.67	45.42 ± 1.77	45.44 ± 1.72	0.687

**Table 4:** Comparison of Sustainable and Healthy Eating Behaviors Scale (SHE) and Nutrition Knowledge Level Scale (NKLSA) Scores according to BMI groups among healthcare workers.

	BMI (kg/m^2^)				
	Underweight (n = 38)	Normal Weight (n = 444)	Overweight (n = 298)	Obesity (n = 290)	*p*
	Mean ± SD	Mean ± SD	Mean ± SD	Mean ± SD	
**SHE subdimensions**					
1. Quality labels (local and organic)	47.45 ± 5.14 ^a,b,c^	35.34 ± 14.80 ^a,d^	29.34 ± 15.79 ^b^	26.46 ± 14.19 ^c,d^	<0.001 *
2. Seasonal foods and avoidance of food waste	41.24 ± 4.49 ^a,b,c^	30.84 ± 12.88 ^a,d^	25.72 ± 13.86 ^b^	23.20 ± 12.44 ^c,d^	<0.001 *
3. Animal welfare	23.71 ± 2.59 ^a,b,c^	17.69 ± 7.32 ^a^	14.70 ± 7.91 ^b^	13.31 ± 7.13 ^c^	<0.001 *
4. Reduced meat consumption	17.87 ± 1.86 ^a,b^	13.25 ± 5.48	11.01 ± 6 ^a^	9.95 ± 5.42 ^b^	<0.001 *
5. Healthy and balanced eating	23.82 ± 2.50 ^a,b,c^	17.67 ± 7.38 ^a^	14.61 ± 7.9 ^b^	13.20 ± 7.13 ^c^	<0.001 *
6. Local food	17.58 ± 1.98 ^a,b,c^	13.13 ± 5.56 ^a^	10.90 ± 5.93 ^b^	9.82 ± 5.34 ^c^	<0.001 *
7. Low-fat consumption	17.71 ± 1.80 ^a,b^	13.11 ± 5.58	10.90 ± 5.92 ^a^	9.77 ± 5.29 ^b^	<0.001 *
Total	189.37 ± 20.07 ^a,b,c,d^	141.02 ± 58.87 ^a,d^	117.18 ± 63.17 ^b^	105.70 ± 56.81 ^c,d^	<0.001 *
**NKLSA**					
**Basic nutrition knowledge**					
Poor	27.23 ± 10.81 ^a^	22.11 ± 14.58	15.19 ± 15.22 ^a^	22.50 ± 14.11	<0.001 *
Moderate	52.72 ± 2.74 ^a^	50.77 ± 2.41	49.65 ± 2.70 ^a^	50.55 ± 2.87	<0.001 *
Good	65 ± 0 ^a^	62.25 ± 2.66	59.33 ± 2.61 ^a^	60.91 ± 3.10	<0.010 *
Very Good	76.44 ± 2.30 ^a,b^	74.08 ± 4.20 ^a^	73.29 ± 4.03 ^b^	70.02 ± 3.15	<0.001 *
**Food preference knowledge**					
Poor	18.20 ± 7.31 ^a,b^	14.56 ± 9.71 ^a^	12.65 ± 11.66 ^b^	15.49 ± 9.70	<0.001 *
Moderate	34.90 ± 1.30 ^a,b^	33.80 ± 1.58 ^a^	33.26 ± 1.62 ^b^	33.74 ± 1.66	<0.001 *
Good	42 ± 0 ^a^	40.38 ± 1.30	39.25 ± 1.42 ^a^	39.91 ± 1.54	0.027 *
Very good	46.72 ± 0.94 ^a,b^	45.91 ± 1.65 ^a^	45.43 ± 1.63 ^b^	44.11 ± 1.37	<0.001 *

*: Significant at the 0.05 level according to one-way analysis of variance. ^a,b,c,d^: Differences between categories with the same superscript letters were significant according to the Tukey HSD post hoc test.

**Table 5:** Distribution of Sustainable and Healthy Eating Behaviors Scale scores and Nutrition Knowledge Level Scale (NKLSA) scores according to anthropometric risk status among healthcare workers.

	Waist Circumference			Neck Circumference			Waist-to-Hip Ratio			Waist-to-Height Ratio		
	No Risk (n = 473)	Risk Present (n = 597)	*p*	No Risk (n = 484)	Risk Present (n = 586)	*p*	No Risk (n = 482)	Risk Present (n = 588)	*p*	No Risk (n = 319)	Risk Present (n = 751)	*p*
	Mean ± SD	Mean ± SD		Mean ± SD	Mean ± SD		Mean ± SD	Mean ± SD		Mean ± SD	Mean ± SD	
**SHE subdimensions**												
1. Quality labels (local and organic)	36.35 ± 14.64	28 ± 15.08	<0.001 *	32.40 ± 15.15	31.11 ± 15.68	0.174	36.29 ± 14.64	27.92 ± 15.07	<0.001 *	34.94 ± 15.01	30.31 ± 15.43	<0.001 *
2. Seasonal foods and avoidance of food waste	31.71 ± 12.73	24.55 ± 13.24	<0.001 *	28.34 ± 13.21	27.19 ± 13.70	0.166	31.66 ± 12.74	24.48 ± 13.23	<0.001 *	30.51 ± 13.10	26.53 ± 13.48	<0.001 *
3. Animal welfare	18.19 ± 7.24	14.05 ± 7.56	<0.001 *	16.28 ± 7.57	15.55 ± 7.80	0.125	18.16 ± 7.25	14.01 ± 7.56	<0.001 *	17.47 ± 7.49	15.21 ± 7.70	<0.001 *
4. Reduced meat consumption	13.63 ± 5.42	10.52 ± 5.75	<0.001 *	12.19 ± 5.70	11.65 ± 5.90	0.126	13.61 ± 5.43	10.49 ± 5.74	<0.001 *	13.08 ± 5.60	11.39 ± 5.83	<0.001 *
5. Healthy and balanced eating	18.19 ± 7.30	13.95 ± 7.56	<0.001 *	16.2 ± 7.59	15.51 ± 7.84	0.143	18.15 ± 7.31	13.91 ± 7.56	<0.001 *	17.46 ± 7.50	15.13 ± 7.73	<0.001 *
6. Local food	13.50 ± 5.49	10.40 ± 5.67	<0.001 *	12.05 ± 5.67	11.54 ± 5.90	0.146	13.48 ± 5.5	10.37 ± 5.66	<0.001 *	12.98 ± 5.63	11.26 ± 5.80	<0.001 *
7. Low-fat consumption	13.49 ± 5.51	10.37 ± 5.65	<0.001 *	12.01 ± 5.68	11.54 ± 5.89	0.181	13.47 ± 5.52	10.34 ± 5.64	<0.001 *	12.98 ± 5.62	11.23 ± 5.79	<0.001 *
Total	145.06 ± 58.21	111.84 ± 60.38	<0.001 *	129.48 ± 60.45	124.08 ± 62.58	0.154	144.83 ± 58.25	111.52 ± 60.34	<0.001 *	139.42 ± 59.82	121.05 ± 61.65	<0.001 *
**NKLSA**												
**Basic nutrition knowledge**												
Poor	27.19 ± 10.78	19.43 ± 15.17	<0.001 *	24.36 ± 13.96	21.10 ± 14.10	0.049 *	27.22 ± 10.80	19.27 ± 15.18	<0.001 *	26.49 ± 11.35	18.95 ± 13.62	<0.001 *
Moderate	52.64 ± 2.75	50.06 ± 2.67	<0.001 *	50.66 ± 2.83	50.44 ± 2.90	0.635	52.71 ± 2.73	50.02 ± 2.65	<0.001 *	50.97 ± 3.51	49.88 ± 3.07	0.129
Good	62.80 ± 2.61	59.33 ± 2.60	0.006 *	60.58 ± 3.02	61.30 ± 3.30	0.602	62.80 ± 2.61	59.33 ± 2.60	0.006 *	62.57 ± 2.94	60.13 ± 2.95	0.095
Very Good	74.36 ± 4.08	71.73 ± 3.99	<0.001 *	73.39 ± 4.40	72.93 ± 4.10	0.194	74.35 ± 4.09	71.70 ± 3.97	<0.001 *	74.29 ± 3.90	72.56 ± 4.30	<0.001 *
**Food preference knowledge**												
Poor	18.19 ± 7.29	13.78 ± 10.61	<0.001 *	16.52 ± 9.51	14.71 ± 9.79	0.106	18.20 ± 7.31	13.71 ± 10.63	<0.001 *	17.52 ± 7.57	12.46 ± 9.06	<0.001 *
Moderate	34.86 ± 1.30	33.47 ± 1.62	<0.001 *	33.69 ± 1.69	33.78 ± 1.63	0.759	34.90 ± 1.29	33.45 ± 1.61	<0.001 *	33.59 ± 2.23	33.05 ± 2.01	0.238
Good	40.70 ± 1.33	39.25 ± 1.42	0.024 *	39.83 ± 1.58	40.00 ± 1.56	0.807	40.70 ± 1.33	39.25 ± 1.42	0.024 *	40.57 ± 1.51	39.60 ± 1.50	0.185
Very good	46.00 ± 1.59	44.81 ± 1.65	<0.001 *	45.54 ± 1.78	45.36 ± 1.67	0.202	46.00 ± 1.60	44.79 ± 1.64	<0.001 *	45.99 ± 1.56	45.17 ± 1.75	<0.001 *

*: Significant at the 0.05 level according to the Student’s *t*-test.

**Table 6:** Distribution of Nutrition Knowledge Level Scale (NKLSA) categories according to BMI groups among healthcare workers.

	Underweight (n = 38)	Normal Weight (n = 444)	Overweight (n = 298)	Obesity (n = 290)	*p*
**NKLSA Category**					
**Basic nutrition knowledge**					
Poor	0 (0.0)	117 (26.4)	101 (33.9)	62 (21.4)	<0.001 *
Moderate	0 (0.0)	35 (7.9)	47 (15.8)	98 (33.8)	
Good	2 (5.3)	8 (1.8)	12 (4.0)	0 (0.0)	
Very good	36 (94.7)	284 (64.0)	138 (46.3)	130 (44.8)	
**Food preference knowledge**					
Poor	0 (0.0)	117 (26.4)	101 (33.9)	62 (21.4)	<0.001 *
Moderate	0 (0.0)	35 (7.9)	47 (15.8)	98 (33.8)	
Good	2 (5.3)	8 (1.8)	12 (4.0)	0 (0.0)	
Very good	36 (94.7)	284 (64.0)	138 (46.3)	130 (44.8)	

*: Significant at the 0.05 level according to the chi-square test.

**Table 7:** Cross-sectional indirect association model between Nutrition Knowledge Level Scale (NKLSA), Sustainable and Healthy Eating Behaviors Scale (SHE), and anthropometric indicators.

Outcome (DV)	Path a (X→M)	Path b (M→Y)	Direct Path c′ (X→Y)	Indirect Effect (a × b)	Total Effect (c)	Result
**BMI**	0.955 **	−1.312 **	1.025 **	−1.254 **	−0.229 **	Suppression pattern
**Waist circumference**	0.955 **	−0.792 **	0.607 **	−0.756 **	−0.149 **	Suppression pattern
**Waist-Hip Ratio**	0.955 **	−0.547 **	0.332 **	−0.523 **	−0.191 **	Suppression pattern
**Waist-Height Ratio**	0.955 **	−0.734 **	0.563 **	−0.701 **	−0.138 **	Suppression pattern
**Neck circumference**	0.955 **	0.051 (ns)	−0.118 (ns)	0.049 (ns)	−0.068 *	No indirect pattern

**: significant at 0.001 level; *: significant at 0.05 level; ns: not significant.

## Data Availability

The data presented in this study are available on request from the corresponding author due to privacy and ethical restrictions.

## Supplementary Materials

The following supporting information can be downloaded at: https://www.mdpi.com/article/10.3390/healthcare14142142/s1.

## Abbreviations

The following abbreviations are used in this manuscript:

BMI	Body Mass Index
CFI	Comparative Fit Index
HC	Hip Circumference
NC	Neck circumference
NCDs	Noncommunicable Diseases
NKLSA	Nutrition Knowledge Level Scale for Adults
RMSEA	Root Mean Square Error of Approximation
SHE	Sustainable and Healthy Eating Behaviors Scale
SRMR	Standardized Root Mean Square Residual
TLI	Tucker–Lewis Index
WC	Waist Circumference
WHR	Waist-to-Hip Ratio
WHtR	Waist-to-Height Ratio
