# Supplementary material for: Are Sustainable and Healthy Eating Behaviors and Levels of Nutritional Knowledge Associated with Obesity and Anthropometric Indicators of Chronic Disease Risk Among Healthcare Workers?
Source: Healthcare (Basel). 2026 Jul 16;14(14):2142. doi: 10.3390/healthcare14142142 (PMC13411917; doi:10.3390/healthcare14142142)
Supplement: Supplementary file 1 [file healthcare-14-02142-s001.zip › healthcare-4371458-supplementary.pdf]

## Supplementary Materials

Supplementary tables supporting data audit, sensitivity analyses, collinearity diagnostics, and cross-sectional indirect association analyses.

**Table S1**

*Theoretical Range Audit for SHE Subscale Scores*

| Variable | Theoretical minimum | Theoretical maximum | n | Zero count | Below minimum count | Above maximum count |
|---|---|---|---|---|---|---|
| Quality labels (local and organic) | 8 | 56 | 1070 | 4 | 80 | 0 |
| Seasonal foods and food waste avoidance | 7 | 49 | 1070 | 5 | 82 | 0 |
| Animal welfare | 4 | 28 | 1070 | 9 | 78 | 0 |
| Reduced meat consumption | 3 | 21 | 1070 | 17 | 71 | 0 |
| Healthy and balanced eating | 4 | 28 | 1070 | 11 | 74 | 0 |
| Local food | 3 | 21 | 1070 | 16 | 75 | 0 |
| Low-fat consumption | 3 | 21 | 1070 | 12 | 72 | 0 |
| SHE total score | 32 | 224 | 1070 | 2 | 88 | 0 |

**Table S2**

*Row-Level Audit of SHE Total and Subscale Scores*

| Total n | Valid SHE total n | Valid SHE subscale and total n | Any theoretical-range issue n | SHE total below 32 n | SHE total zero n | SHE total equals subscale sum n | SHE total not equal to subscale sum n |
|---|---|---|---|---|---|---|---|
| 1070 | 982 | 982 | 88 | 88 | 2 | 1070 | 0 |

**Table S3**

*Original and Theoretical-Range-Screened SHE Datasets*

| Dataset | n | SHE issue rows remaining | SHE total below 32 remaining | SHE total zero remaining | SHE total mean | SHE total SD | SHE total minimum | SHE total maximum |
|---|---|---|---|---|---|---|---|---|
| Original dataset | 1070 | 88 | 88 | 2 | 126.53 | 61.655 | 0 | 223 |
| Theoretical-range-screened SHE dataset | 982 | 0 | 0 | 0 | 136.38 | 54.351 | 32 | 223 |
| Theoretical-range-screened SHE subscale and total dataset | 982 | 0 | 0 | 0 | 136.38 | 54.351 | 32 | 223 |

**Table S4**

*Key Correlations Between NKLSA, SHE, and Anthropometric Indicators*

| Dataset | X variable | Y variable | n | Pearson r | 95% CI lower | 95% CI upper | p |
|---|---|---|---|---|---|---|---|
| Original dataset | NKLSA total score | SHE total score | 1070 | 0.955 | 0.95 | 0.96 | <0.001 |

| Dataset | X variable | Y variable | n | Pearson r | 95% CI lower | 95% CI upper | p |
|---|---|---|---|---|---|---|---|
| Original dataset | SHE total score | BMI | 1070 | -0.333 | -0.386 | -0.279 | 3.30e-29 |
| Original dataset | SHE total score | Waist circumference | 1070 | -0.211 | -0.268 | -0.153 | 2.94e-12 |
| Original dataset | SHE total score | Neck circumference | 1070 | -0.061 | -0.12 | -0.001 | 0.046 |
| Original dataset | SHE total score | Waist-to-hip ratio | 1070 | -0.23 | -0.286 | -0.172 | 2.70e-14 |
| Original dataset | SHE total score | Waist-to-height ratio | 1070 | -0.196 | -0.253 | -0.138 | 9.91e-11 |
| Theoretical-range-screened SHE dataset | NKLSA total score | SHE total score | 982 | 0.948 | 0.941 | 0.954 | <0.001 |
| Theoretical-range-screened SHE dataset | SHE total score | BMI | 982 | -0.29 | -0.346 | -0.231 | 1.84e-20 |
| Theoretical-range-screened SHE dataset | SHE total score | Waist circumference | 982 | -0.159 | -0.219 | -0.097 | 5.79e-07 |
| Theoretical-range-screened SHE dataset | SHE total score | Neck circumference | 982 | -0.009 | -0.072 | 0.053 | 0.775 |
| Theoretical-range-screened SHE dataset | SHE total score | Waist-to-hip ratio | 982 | -0.148 | -0.209 | -0.086 | 3.08e-06 |
| Theoretical-range-screened SHE dataset | SHE total score | Waist-to-height ratio | 982 | -0.14 | -0.2 | -0.078 | 1.12e-05 |

**Table S5**

*Collinearity Diagnostics for Models Including NKLSA and SHE*

| Dataset | Variable | VIF | Interpretation |
|---|---|---|---|
| Original dataset | NKLSA total score | 11.535 | Elevated |
| Original dataset | SHE total score | 11.536 | Elevated |
| Theoretical-range-screened SHE dataset | NKLSA total score | 9.892 | Borderline |
| Theoretical-range-screened SHE dataset | SHE total score | 9.891 | Borderline |
| Theoretical-range-screened SHE subscale and total dataset | NKLSA total score | 9.892 | Borderline |
| Theoretical-range-screened SHE subscale and total dataset | SHE total score | 9.891 | Borderline |

**Table S6**

*Cross-Sectional Indirect Association and Suppression-Pattern Diagnostics*

| Dataset | Outcome | n | Path a (X to M) | Path b (M to Y, adjusted) | Direct path c′ | Indirect path a×b | Total path c | Pattern |
|---|---|---|---|---|---|---|---|---|
| Original dataset | BMI | 1070 | 1.574 | -0.114 | 0.146 | -0.179 | -0.032 | Inconsistent indirect association/suppression pattern |
| Original dataset | Waist circumference | 1070 | 1.574 | -0.22 | 0.275 | -0.346 | -0.071 | Inconsistent indirect association/suppression pattern |
| Original dataset | Neck circumference | 1070 | 1.574 | 0.003 | -0.012 | 0.005 | -0.008 | Inconsistent indirect association/suppression |

| Dataset | Outcome | n | Path a (X to M) | Path b (M to Y, adjusted) | Direct path c′ | Indirect path a×b | Total path c | Pattern |
|---|---|---|---|---|---|---|---|---|
| | | | | | | | | pattern |
| Original dataset | Waist-to-hip ratio | 1070 | 1.574 | -0.001 | 0.001 | -0.002 | -0.001 | Inconsistent indirect association/suppression pattern |
| Original dataset | Waist-to-height ratio | 1070 | 1.574 | -0.001 | 0.001 | -0.002 | 0.000 | Inconsistent indirect association/suppression pattern |
| Theoretical-range-screened SHE dataset | BMI | 982 | 1.747 | -0.144 | 0.224 | -0.251 | -0.027 | Inconsistent indirect association/suppression pattern |
| Theoretical-range-screened SHE dataset | Waist circumference | 982 | 1.747 | -0.3 | 0.482 | -0.523 | -0.042 | Inconsistent indirect association/suppression pattern |
| Theoretical-range-screened SHE dataset | Neck circumference | 982 | 1.747 | -0.005 | 0.008 | -0.008 | 0.000 | Inconsistent indirect association/suppression pattern |
| Theoretical-range-screened SHE dataset | Waist-to-hip ratio | 982 | 1.747 | -0.002 | 0.003 | -0.004 | 0.000 | Inconsistent indirect association/suppression pattern |
| Theoretical-range-screened SHE dataset | Waist-to-height ratio | 982 | 1.747 | -0.002 | 0.003 | -0.003 | 0.000 | Inconsistent indirect association/suppression pattern |

**Table S7**

*Verification of Anthropometric Risk Cut-Off Values and Risk Counts*

| Measure | Sex | Variable | Risk cut-off | n | Mean | SD | Risk-present n |
|---|---|---|---|---|---|---|---|
| Waist circumference | Female | Waist circumference | >=80 | 523 | 83.647 | 13.995 | 287 |
| Waist circumference | Male | Waist circumference | >=94 | 547 | 97.574 | 14.818 | 310 |
| Neck circumference | Female | Neck circumference | >=33 | 523 | 35.992 | 4.388 | 377 |
| Neck circumference | Male | Neck circumference | >=37 | 547 | 35.913 | 4.182 | 209 |
| Waist-to-hip ratio | Female | Waist-to-hip ratio | >=0.85 | 523 | 0.863 | 0.143 | 283 |
| Waist-to-hip ratio | Male | Waist-to-hip ratio | >=0.90 | 547 | 0.92 | 0.139 | 305 |
| Waist-to-height ratio | Both | Waist-to-height ratio | <0.4 or >=0.5 | 1070 | 0.548 | 0.093 | 751 |

**Table S8**

*ANOVA Effect Sizes for BMI-Group Comparisons*

| Variable | n | F | p | η² | ω² | Levene p |
|---|---|---|---|---|---|---|
| Quality labels (local and organic) | 1070 | 38.465 | <0.001 | 0.098 | 0.095 | <0.001 |
| Seasonal foods and food waste avoidance | 1070 | 37.127 | <0.001 | 0.095 | 0.092 | <0.001 |
| Animal welfare | 1070 | 37.944 | <0.001 | 0.096 | 0.094 | <0.001 |
| Reduced meat consumption | 1070 | 38.161 | <0.001 | 0.097 | 0.094 | <0.001 |
| Healthy and balanced eating | 1070 | 39.369 | <0.001 | 0.100 | 0.097 | <0.001 |

| Variable | n | F | p | $\eta^2$ | $\omega^2$ | Levene p |
|---|---|---|---|---|---|---|
| Local food | 1070 | 37.443 | <0.001 | 0.095 | 0.093 | <0.001 |
| Low-fat consumption | 1070 | 38.680 | <0.001 | 0.098 | 0.096 | <0.001 |
| SHE total score | 1070 | 38.275 | <0.001 | 0.097 | 0.095 | <0.001 |
| Basic nutrition knowledge | 1070 | 23.593 | <0.001 | 0.062 | 0.060 | <0.001 |
| Food preference knowledge | 1070 | 20.186 | <0.001 | 0.054 | 0.051 | <0.001 |
| NKLSA total score | 1070 | 22.296 | <0.001 | 0.059 | 0.056 | <0.001 |

## Table S9

*Two-Group Comparisons by Anthropometric Risk Status With Cohen's d*

| Grouping variable | Variable | Group 1 | n 1 | Mean 1 ± SD | Group 2 | n 2 | Mean 2 ± SD | p | Cohen's d |
|---|---|---|---|---|---|---|---|---|---|
| Waist circumference risk status | SHE total score | No risk | 473 | 145.06±58.21 | Risk present | 597 | 111.84±60.38 | <0.001 | 0.559 |
| Waist circumference risk status | Basic nutrition knowledge | No risk | 473 | 61.03±21.15 | Risk present | 597 | 50.97±23.99 | <0.001 | 0.441 |
| Waist circumference risk status | Food preference knowledge | No risk | 473 | 38.21±12.55 | Risk present | 597 | 32.51±14.82 | <0.001 | 0.411 |
| Waist circumference risk status | NKLSA total score | No risk | 473 | 99.24±33.68 | Risk present | 597 | 83.49±38.79 | <0.001 | 0.430 |
| Neck circumference risk status | SHE total score | No risk | 484 | 129.48±60.45 | Risk present | 586 | 124.08±62.58 | 0.153 | 0.088 |
| Neck circumference risk status | Basic nutrition knowledge | No risk | 484 | 56.59±22.33 | Risk present | 586 | 54.45±24.07 | 0.132 | 0.092 |
| Neck circumference risk status | Food preference knowledge | No risk | 484 | 35.76±13.46 | Risk present | 586 | 34.42±14.67 | 0.120 | 0.095 |
| Neck circumference risk status | NKLSA total score | No risk | 484 | 92.36±35.77 | Risk present | 586 | 88.87±38.71 | 0.127 | 0.093 |
| Waist-to-hip ratio risk status | SHE total score | No risk | 482 | 144.83±58.25 | Risk present | 588 | 111.52±60.34 | <0.001 | 0.561 |
| Waist-to-hip ratio risk status | Basic nutrition knowledge | No risk | 482 | 60.94±21.18 | Risk present | 588 | 50.89±24.01 | <0.001 | 0.441 |
| Waist-to-hip ratio risk status | Food preference knowledge | No risk | 482 | 38.16±12.57 | Risk present | 588 | 32.46±14.84 | <0.001 | 0.411 |
| Waist-to-hip ratio risk status | NKLSA total score | No risk | 482 | 99.11±33.72 | Risk present | 588 | 83.35±38.83 | <0.001 | 0.430 |
| Waist-to-height ratio risk status | SHE total score | No risk | 319 | 139.42±59.82 | Risk present | 751 | 121.05±61.65 | <0.001 | 0.301 |
| Waist-to-height ratio risk status | Basic nutrition knowledge | No risk | 319 | 59.32±21.81 | Risk present | 751 | 53.76±23.74 | <0.001 | 0.240 |
| Waist-to-height ratio risk status | Food preference knowledge | No risk | 319 | 37.25±13.00 | Risk present | 751 | 34.09±14.51 | <0.001 | 0.224 |
| Waist-to-height ratio risk status | NKLSA total score | No risk | 319 | 96.57±34.79 | Risk present | 751 | 87.85±38.22 | <0.001 | 0.234 |

**Table S10**

*NKLSA Category Distributions by Anthropometric Risk Status*

| Level variable | Grouping variable | Level | No risk n (%) | Risk present n (%) | p |
|---|---|---|---|---|---|
| Basic nutrition knowledge | Waist circumference | Poor | 114 (24.1) | 166 (27.8) | <0.001 |
| Basic nutrition knowledge | Waist circumference | Moderate | 34 (7.2) | 146 (24.5) | |
| Basic nutrition knowledge | Waist circumference | Good | 10 (2.1) | 12 (2.0) | |
| Basic nutrition knowledge | Waist circumference | Very good | 315 (66.6) | 273 (45.7) | |
| Basic nutrition knowledge | Neck circumference | Poor | 118 (24.4) | 162 (27.6) | 0.503 |
| Basic nutrition knowledge | Neck circumference | Moderate | 86 (17.8) | 94 (16.0) | |
| Basic nutrition knowledge | Neck circumference | Good | 12 (2.5) | 10 (1.7) | |
| Basic nutrition knowledge | Neck circumference | Very good | 268 (55.4) | 320 (54.6) | |
| Basic nutrition knowledge | Waist-to-hip ratio | Poor | 117 (24.3) | 163 (27.7) | <0.001 |
| Basic nutrition knowledge | Waist-to-hip ratio | Moderate | 35 (7.3) | 145 (24.7) | |
| Basic nutrition knowledge | Waist-to-hip ratio | Good | 10 (2.1) | 12 (2.0) | |
| Basic nutrition knowledge | Waist-to-hip ratio | Very good | 320 (66.4) | 268 (45.6) | |
| Basic nutrition knowledge | Waist-to-height ratio | Poor | 84 (26.3) | 196 (26.1) | <0.001 |
| Basic nutrition knowledge | Waist-to-height ratio | Moderate | 29 (9.1) | 151 (20.1) | |
| Basic nutrition knowledge | Waist-to-height ratio | Good | 7 (2.2) | 15 (2.0) | |
| Basic nutrition knowledge | Waist-to-height ratio | Very good | 199 (62.4) | 389 (51.8) | |
| Food preference knowledge | Waist circumference | Poor | 114 (24.1) | 166 (27.8) | <0.001 |
| Food preference knowledge | Waist circumference | Moderate | 34 (7.2) | 146 (24.5) | |
| Food preference knowledge | Waist circumference | Good | 10 (2.1) | 12 (2.0) | |
| Food preference knowledge | Waist circumference | Very good | 315 (66.6) | 273 (45.7) | |
| Food preference knowledge | Neck circumference | Poor | 118 (24.4) | 162 (27.6) | 0.503 |
| Food preference knowledge | Neck circumference | Moderate | 86 (17.8) | 94 (16.0) | |
| Food preference knowledge | Neck circumference | Good | 12 (2.5) | 10 (1.7) | |
| Food preference knowledge | Neck circumference | Very good | 268 (55.4) | 320 (54.6) | |
| Food preference knowledge | Waist-to-hip ratio | Poor | 117 (24.3) | 163 (27.7) | <0.001 |
| Food preference knowledge | Waist-to-hip ratio | Moderate | 35 (7.3) | 145 (24.7) | |
| Food preference knowledge | Waist-to-hip ratio | Good | 10 (2.1) | 12 (2.0) | |
| Food preference knowledge | Waist-to-hip ratio | Very good | 320 (66.4) | 268 (45.6) | |
| Food preference knowledge | Waist-to-height ratio | Poor | 84 (26.3) | 196 (26.1) | <0.001 |
| Food preference knowledge | Waist-to-height ratio | Moderate | 29 (9.1) | 151 (20.1) | |
| Food preference knowledge | Waist-to-height ratio | Good | 7 (2.2) | 15 (2.0) | |
| Food preference knowledge | Waist-to-height ratio | Very good | 199 (62.4) | 389 (51.8) | |
